# Linking Colorimetric Variation with Non-Volatile and Volatile Components of Carob Flour

**DOI:** 10.3390/foods12132556

**Published:** 2023-06-30

**Authors:** Chrystalla Antoniou, Marios C. Kyriacou, Angelos C. Kyratzis, Youssef Rouphael

**Affiliations:** 1 Department of Vegetable Crops, Agricultural Research Institute, Nicosia 1516, Cyprus; chrysanto90@gmail.com (C.A.); akyratzis@ari.moa.gov.cy (A.C.K.); 2 Department of Agricultural Sciences, University of Naples Federico II, 80055 Portici, Italy

**Keywords:** antioxidants, *Ceratonia siliqua* L., color, HS-SPME GC-MS, phenols, sugars

## Abstract

Chromatic variation was examined for its association with flour composition and quality. Carob samples from variable altitudes and genetic backgrounds were milled and assessed for colorimetric parameter L* (lightness) and analyzed for phenols, tannins, antioxidant capacity, soluble carbohydrates (HPLC-RID), organic acids and protein (IC-CD), and volatile organic compounds (VOCs; HS-SPME/GC-MS). Higher altitudes and grafted genotypes yielded lighter-colored flours of higher antioxidant potential, phenols, tannins, sucrose, and malic acid concentrations. VOCs were mainly acids, esters, aldehydes, ketones, and alcohols. Acids were the most abundant and correlated negatively with L*, though correlation for many individual acids was non-significant, including 2-methyl-propanoic acid, widely considered the carob signature aroma (cheesy acidic buttery). The compositional and quality indexing potential of L* is more robust for grafted than non-grafted material, owing putatively to a narrower genetic basis. Antioxidant capacity and concentrations of phenolics, tannins and sucrose correlated positively with L*, indicating increased levels in carob flours sourced from grafted trees at higher altitudes. These flours also have a lower content of reducing sugars, the implication of which in the darkening of carob flour warrants further investigation. Overall, L* constitutes a reliable index for ranking carob flours for key compositional attributes and may be further reinforced by multiple-year data.

## 1. Introduction

Renewed global interest in the cultivation of the carob tree (*Ceratonia siliqua* L.), formerly confined to the Mediterranean basin, stems in part from the superior resilience of this leguminous species to harsh edaphoclimatic conditions and limited input requirements relative to other fruit tree crops [1,2,3]. The ensuing climate change and the growing threat to food security have thus favored the expansion of the carob crop, especially to marginalized agricultural lands, such as impoverished calcareous soils receiving limited precipitation. Moreover, the expansion of the carob crop has been driven by growing industrial demand for the pod and its byproducts.

The seed-to-pulp weight ratio in mature carob pods ranges from 5 to 15, with higher ratios observed in wild genotypes [2]. The carob seed endosperm is the source of the Locust Bean Gum (LBG), a natural food gelling agent consisting chiefly of galactomannan polymers, the demand for which has soared in recent years [4]. However, the kibbled pulp has recently attracted growing market demand as well, though formerly it was regarded chiefly as a low-cost by-product of the milling process destined for livestock feed. The pulp of the mature carob is rich in sugars (sucrose > fructose > glucose) and cyclitols but also in dietary fibers, polyphenols, and minerals, especially potassium, as opposed to low lipid content [2,5,6]. Kibbled carob pulp is mainly ground to carob flour or extracted for sugars and subsequent condensation to molasses. Further to its lower cost and ecofriendly profile, carob flour also poses certain nutritive advantages over cocoa, including lower fat content, higher dietary fiber content and the absence of caffeine and theobromine [7,8]. Carob flour is currently used also in breadmaking, confectionery and pasta formulations [9,10].

Recent work has demonstrated wide variation in the morphological and compositional traits of the carob pod, governed by genotype, environment (proximity to the sea and altitude), and seasonal variation expressed in alternate bearing cycles [2]. For instance, pod dimensions, seed-to-pulp ratio, and pulp total sugar content seem primarily under genetic control, whereas the agro-environmental conditions have a strong effect on phenolic content, antioxidant activity, condensed tannins, reducing sugars/sucrose ratio and the color of the carob pulp. Catechins, hydrolyzable tannins, and flavone glycosides concentrate more in the pulp of carobs growing at higher altitudes (300–700 m; Kyriacou et al. [6]). It is therefore evident that the quality and composition of carob flour will reflect that of the source material milled. Significant variation was similarly observed with respect to the profile of volatile organic compounds (VOCs) responsible for the aroma of the carob flour [11]. Volatile acids associated with less pleasant aroma were more abundant in carobs obtained from lower altitudes near coastal regions; conversely, the relative abundance of isobutyric acid (responsible for the cheesy acidic buttery aroma trait of carob) and the overall sensory quality of the carob flour were enhanced in kibbles sourced from higher altitudes. An additional source of potential variation in the quality and composition of carob flour is the maturity of the pods at harvest. For instance, catechins and hydrolyzable tannins dominate the immature green stages of the pod, whereas the onset of physiological maturity signals a sharp decline in catechins, hydrolyzable gallotannins, and flavonol glycosides, manifested also in reduced antioxidant capacity [6].

Roasting of the carob kibbles is a common practice aiming at reducing residual moisture and obtaining a uniformly powdered product [12]. It is usually performed at around 150 °C for 10 to 60 min [13]. Variation in roasting temperature and duration results in carob flour of variable grades. Roasting increased the concentration of phenolic compounds and tannins in carob flour, while it also increased the antioxidant capacity owing likely to the higher solubility of phenolic compounds [13,14]. However, roasting also increased the browning index of the flour and reduced the concentration of isobutyric acid in the kibbles depending on roasting temperature and duration [15]. Sugar caramelization and Maillard reaction, which take place during the roasting of carob kibbles, impact significantly the quality and composition of the final product [16]. Although the roasting process tends to homogenize the quality of flour produced from kibbles of variable quality and masks any defects associated with the raw material, it is also responsible for the increase in Maillard reaction products in the carob flour, such as furfural and hydroxymethyl-2-furaldehyde, especially when temperatures exceed 130 °C [7]. The analysis of carob samples roasted at different temperatures (100–180 °C) also showed that the level of carcinogenic polycyclic aromatic hydrocarbons (PAHs) increased in roasted samples with temperature relative to the unroasted control [14]. Granted the above considerations, lyophilization constitutes a non-thermal alternative to roasting capable of reducing kibble moisture content, thus easing the milling process and stabilizing the end product. Notwithstanding its higher cost, lyophilization presents several advantages over roasting, such as retention of nutrients, color, and aroma, as well as more porous texture and improved rehydration potential [17].

The retention of traits reflecting the quality of the raw material further enables the potential assessment of quality across different grades of raw carob pulp and carob flour. Instrumental non-destructive quantification of key quality traits is the key to the standardization and monitoring of quality along the supply chain of carob raw material and carob flour, [18]. Developing quality standards for carob flour based on non-destructive methods of assessment, such as reflectance colorimetry, will undeniably support the promotion of carob trade, import–export activities, and the restriction of low-quality products. Numerous previous studies have highlighted the importance of developing quality standards to ensure product consistency, satisfaction consumers’ and supply chain stakeholders’ expectations, and the development of added value products based on robust preselection of quality raw material [18,19,20]. In this perspective, the current study aimed at investigating the association of color with the non-volatile and volatile components of carob flours milled from lyophilized, kibbled carob pods obtained at full maturity from genetically identified grafted and non-grafted trees located in variable agro-environments. Carob flours milled from sampled pods were subjected to reflectometric assessment of colorimetric parameter L* (lightness) and compositional analyses, including spectroscopic analysis of total phenolic compounds, tannins, and in vitro antioxidant capacity, HPLC of soluble sugars, ion chromatography of organic acids and protein, and HS-SPME VOCs isolation and GC-MS analysis. The current work introduces the colorimetric prognostication of carob flour composition, volatilomic, and functional quality attributes as a novel tool for application in the supply chain of carob raw material and carob flour toward the standardization of quality.

## 2. Materials and Methods

### 2.1. Plant Material and Sample Preparation

Carob fruits were harvested at commercial maturity stage RS5 [6], from 68 grafted trees, exhibiting narrow genetic variability and representing the main local landrace, and 13 non-grafted genetically diverse trees, all georeferenced, ranging in habitat altitude from 15 m in the coastal zone to 510 m in the mountainous inland [2]. Sampling was repeated for two years (2018 and 2019). Samples consisting of 30–40 carob pods were harvested from singular, identified, georeferenced trees. Harvest was performed in the morning hours (08:00–10:00) and the samples were directly transferred to the Postharvest Physiology Laboratory of the Agricultural Research Institute where pods were coarsely ground in a Vita Prep 3 blender (Vita-Mix Corp., Cleveland, OH, USA) operated at low speed and deseeded. Coarse kibbles were frozen at −40 °C and lyophilized at 0 °C for 48 h in a Christ, Alpha 1–4 lyophilizer (Osterode, Germany). Fully desiccated kibbles were ground in a CT293 Cyclotech mill (Foss Analytical A/S, Hillerød, Denmark) to pass through a 1 mm sieve and the flour obtained was stored at −60 °C for compositional analyses. 

### 2.2. Carob Flour Colorimetry

**Figure 1:**
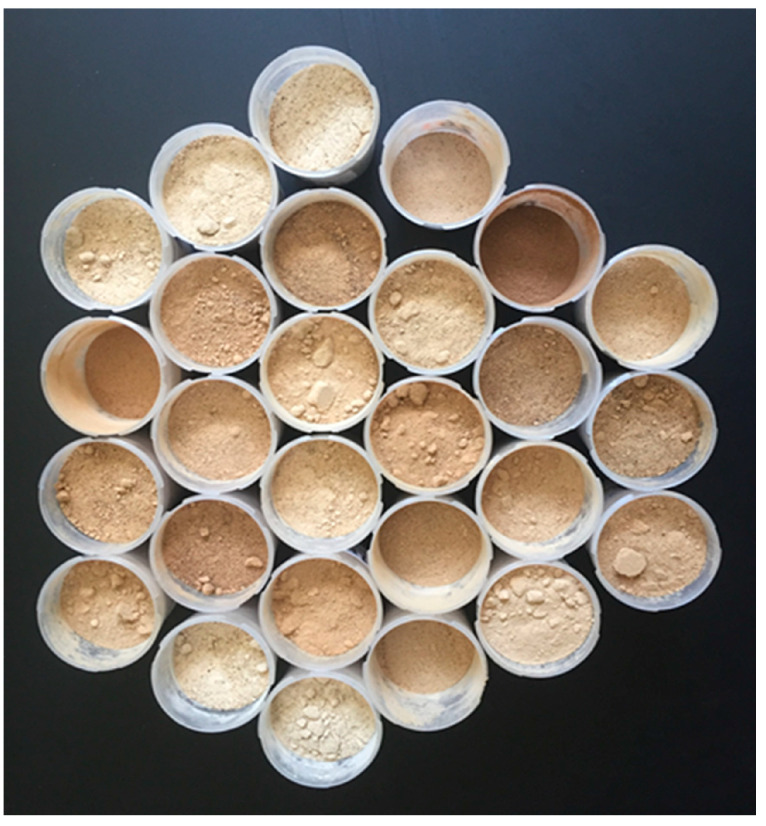
Optical variation in color among samples of carob flours milled from lyophilized carob pulp sourced from variable agro-environments and genotypes.

The color of the carob flour was determined using a Minolta CR-410 Chroma Meter (Minolta, Osaka, Japan) equipped with a diffusion illumination 0° viewing angle geometry and the color space XYZ, Yxy, L*a*b*, Hunter, L*C*h, Munsel as the default [21]. The color component L* (lightness), which ranges from 0 = black to 100 = white, was recorded as the variable expressing the darkness of carob flour samples (Figure 1).

### 2.3. Carob Flour Concentration in Total Phenolic Compounds and Tannins

The carob flour total phenolic content (TPC) was extracted in methanol:H_2_O:acetate (50:49.5:0.5) for 24 h at 4 °C in the dark and quantified based on the modification of Singleton et al. [22], described previously by Kyriacou et al. [6]. Quantification was based on the absorbance at 765 nm determined on a Jasco V-550 spectrometer (Jasco Corp., Tokyo, Japan) against linear calibration (R^2^ > 0.99) with gallic acid (50−500 mg L^−1^) as an external standard and the results were denoted as g kg^−1^ flour dw in gallic acid equivalents. 

Carob flour content in condensed tannins (proanthocyanidins) was determined according to a modification of the vanillin methods of Sun et al. [23] and Sepperer et al. [24], described previously by Antoniou et al. [25]. Lyophilized flour 0.5 g was extracted in 10 mL methanol:H_2_O:HCl (50:40:10) at 4 °C for 24 h in the dark. Quantification was performed at 500 nm against calibration with catechin external standards (0.025–0.5% *w*/*v* in methanol), and the results were expressed in mg catechin equivalents (CE) per g dw.

### 2.4. Carob Flour Radical Scavenging Capacity and Antioxidant Potential

The ascorbate equivalent antioxidant activity (AEAC) of carob flour methanolic extracts was assayed using the 1,1-diphenyl-2-picrylhydrazyl free-radical (DPPH) scavenging capacity in vitro assay [26]. Quantification was facilitated by a calibration curve established with ascorbate external standards (100–1000 µM), and results were expressed in ascorbate equivalents (AAE) as g AAΕ kg^−1^ dw. Methanolic extracts were also assayed in vitro for ferric reducing antioxidant power (FRAP), based on the method of Benzie and Strain [26]. External standard calibration curves were established using ascorbic acid concentrations of 100–1000 μΜ, and the results were expressed in ascorbate equivalents as g AAΕ kg^−1^ dw.

### 2.5. Sugars, Organic Acids, and Protein Concentrations in Carob Flour

Water-soluble sugars were analyzed on aqueous extracts of carob flour following clarification using the Carrez Clarification Kit (Sigma-Aldrich, St. Louis, MO, USA), as previously described [25]. Fructose, glucose, and sucrose comprised the principle water-soluble carbohydrates separated and identified by liquid chromatography on an Agilent 1260 Series HPLC-RID system (Agilent Technologies, Santa Clara, CA, USA). Sugars were separated on a Waters 4.6 × 250 mm carbohydrate column (Waters, Milford, MA, USA) at 35 °C. Acetonitrile:water (82:18) was used as the mobile phase at an isocratic flow rate of 1.5 mL min^−1^. The injection volume was 20 μL, and calibration curves for fructose, glucose, and sucrose external standards (0.2–2.0 g 100^−1^ mL) were established with coefficients of determination R^2^ > 0.999. Results were expressed as g 100 g^−1^ dw.

Analysis of organic acids in carob flour was performed as described by Rouphael et al. [27] on a Dionex ICS-3000 ion chromatograph (Dionex, Sunnyvale, CA, USA) equipped with an electrical conductivity detector. Organic acids were separated through an IonPac AS11-HC analytical column (4 × 250 mm) guarded by an IonPac AG11-HC (4 × 50 mm) column. The concentrations of organic acids were reported as mg g^−1^ dw.

The nitrogen content of carob flour was determined by the Kjeldahl method and the total protein content of carob flour was estimated using a nitrogen-to-protein conversion factor of 6.25 [28].

### 2.6. HS-SPME Volatiles Isolation and GC-MS Analysis of Volatile Organic Compounds (VOCs) in Carob Flour

#### 2.6.1. HS-SPME Isolation of VOCs

Isolation of the carob flour headspace volatile fraction was facilitated using a 2 cm Supelco (Park, Bellefonte, PA, USA) fiber coated with 50/30 μm Divinylbenzene/Carboxen/Polydimethylsiloxane (DVB/CAR/PDMS). About four grams of carob flour from each sample was weighed directly into 20 mL clear glass vials (Agilent Technologies, Santa Clara, CA, USA), which were immediately crimp-sealed with 20 mm caps and incubated for 24 h at 25 °C. Before adsorption, the emission of VOCs was enhanced by raising the incubation temperature to 50 °C for 30 min [29]. Subsequently, the SPME fiber was inserted through the cap septum into the vial and exposed for 30 min to the headspace of the sample to adsorb VOCs. Samples for each genotype, location, and ripening stage were analyzed in triplicate. The SPME fiber was preconditioned at 300 °C for 30 min before each run and a system blank injection was performed between samples to minimize contamination and detect carryover residual compounds.

#### 2.6.2. Analysis of VOCs by Gas Chromatography-Mass Spectrometry

Analysis of VOCs was executed on an Agilent 7890A GC system, connected to a 5977B GC/MSD mass selective detector (Agilent Technologies, Santa Clara, CA, USA). A Supelco SPB-624 capillary column of 60 m × 0.25 mm with 1.40 μm film thickness (fused silica) facilitated the separation of compounds. Desorption of adsorbed compounds was thermally facilitated at 280 °C by exposing the SPME fiber for 1 min into the GC inlet operated in a 1:10 split mode. The methodology of Krokou et al. [30] was followed for setting the GC conditions: inlet temperature was 280 °C and oven temperature started at 35 °C for 5 min, ramped to 180 °C at 4 °C/min, and kept for 10 min. A continuous flow of helium at the rate of 1.7 mL min^−1^ was used as a carrier. The mass spectrometer was operated within the spectra range of 35 to 350 *m*/*z* in the electron ionization mode (EI) at 70 eV. The MS source, transfer line, and quadrupole temperatures were 230 °C, 250 °C, and 150 °C, respectively. All biological replicates were extracted and analyzed in parallel under identical conditions. For the identification of VOCs, recorded mass spectra were matched with those stored in the NIST17 library of the GC-MS data system. Verification was further conducted by comparison of retention indices with external analytical standards, including the volatile free acid mixture (Supelco, CRM46975), butyric acid (Sigma-Aldrich, W222208), isobutyric acid, (Sigma-Aldrich, W222119), valeric acid (Sigma-Aldrich, W310107), and acetic acid (Sigma-Aldrich, 695092). The percentage quantitation was based on peak area normalization without using correction factors.

### 2.7. Statistical Analysis

Means, maximum, minimum values, and standard errors of compositional and functional traits are presented. A t-test was employed to investigate significant differences between grafted and non-grafted trees and between years. Pearson correlations were estimated to investigate the relations between years for each trait. The relations between flour lightness (L*) with traits were investigated employing Pearson correlations. Regression analysis was performed when correlations were high to obtain a prediction model based on flour lightness. The equality of the slopes and of the intercepts was tested according to [31]. Stepwise regressions were conducted to model the traits that contribute significantly to the variation in flour lightness. The correlation and regression analyses were performed separately in data originating from grafted and non-grafted trees and different years because of the different genetic basis and the significant variation between years, respectively. Correlations were also calculated on data derived from all trees collectively within each year to reflect the variation in the raw material received by the carob mills, which is a mixture of pods from grafted and non-grafted trees. Principal component analysis (PCA) was employed to depict relations between variables and the origin of the trees. Biplots were constructed for each year. 

Means and standard errors of the relative abundance of VOCs were calculated. Pearson correlations were estimated to investigate the relationship between flour lightness (L*) with VOCs. The t-test was applied to investigate the significance of differences in VOCs between grafted and non-grafted trees. The correlation analysis was performed on pooled data derived from grafted and non-grafted trees because the differences between the two groups were non-significant for most of the volatile compounds identified and quantitated. All statistical analyses were performed using SPSS statistical package (IBM, SPSS ver. 25).

## 3. Results and Discussion

### 3.1. Carob Flour Colorimetry

Carob flour colorimetry was assessed in terms of CIELAB color parameter L*-lightness [6]. The annual means of L* for the 2018 and 2019 harvest seasons were relatively stable for both the grafted and non-grafted material sampled (Table 1 and Table 2). Grafted material yielded lighter and less variable flour color than non-grafted material (L* = 68.05–69.75 vs. L* = 63.38–63.54) in both years of the study, which likely reflects its narrower genetic basis and cumulative selection pressure for improved phenotypes, compared to the non-grafted material [2]. Previous work has indicated the prevailing differentiation of grafted to non-grafted carob stock as well as the influential role of agro-environmental zones on pod phytochemical characteristics [32,33]. A recent work has also showed that pod pulp color is also defined by the genetic material and agro-enviromental zone chiefly by altitude and proximity to the sea [6]. The current work corroborates these findings, as lighter pulp color and subsequently lighter-colored carob flour was obtained from unroasted pods sourced from inland populations of grafted carob trees (Table 3).

### 3.2. Radical Scavenging Capacity and Antioxidant Potential of Carob Flour

The appraisal of antioxidant capacity for carob flour methanolic extracts indicated that both the radical scavenging capacity (DPPH) and the ferric reduction antioxidant potential (FRAP) in vitro assays yielded higher mean values for the non-grafted material in both years of the study (Table 1 and Table 2). However, this difference was statistically significant only for the DPPH scores of 2018 due to the high variance of values observed in both the grafted and non-grafted source material. This wide variation confounds the potential differentiation of the source material based on genotype. It may be explained, however, based on previous findings that the antioxidant capacity of the carob fruit pulp is extensively influenced by the agro-environmental conditions prevailing in the zone of cultivation, with the major divide being low vs. high altitude [2]. This is further reinforced by the present results showing a significant correlation of yearly values for DPPH and FRAP despite significant differentiation in yearly means.

### 3.3. Carob Flour Content in Total Phenolic Compounds and Tannins

The content of carob flour in total phenolic compounds demonstrated a similar pattern to that observed for the in vitro antioxidant capacity assays (Table 1 and Table 2). The mean phenolic content in carob flour from non-grafted material was higher than that obtained from grafted material by 19.8% in 2018 and 35.2% in 2019. However, these mean values were not significantly different due to the variance introduced mainly by the variation in the agro-environmental conditions prevailing at the sampling sites. In the case of tannins, which constitute phenolic macromolecules, grafted and non-grafted material exhibited very similar mean values in both sampling years. It is further worth noting that the phenolics and tannins concentrations in the flour obtained from grafted material in the two sampling years correlated significantly, whereas the annual mean values for the non-grafted material did not, which likely reflects the wider genetic basis and smaller sample size of the non-grafted carob population. Previous work has also indicated that the roasting process affects the polyphenols content of flour carob flour, attributed mainly to the degradation of flavanol glycosides and concomitant increase in the relative levels of aglycones [34].

### 3.4. Carob Flour Content in Sugars, Organic Acids, and Protein

The total sugar content of carob flour differed significantly between sampling years (Table 1 and Table 2). Carob flour sugar content varied similarly between years for both grafted and non-grafted material, being higher in 2018 by 17.0% and 18.0% in grafted and non-grafted material, respectively. This rise and drop in the sugar content of the carob flour is closely related to the phenomenon of the alternate bearing of the carob tree, as highlighted in previous works [2]. It is also worth noting that the total sugars content of carob flour was higher in grafted material by 13.3% and 14.3% in 2018 and 2019, respectively. (Table 1). Differences in total sugars content between grafted and non-grafted materials derived mainly from sucrose levels, which comprised 71.8% and 68.0% of the mean total sugars content in grafted and non-grafted material for 2018 and 2019, respectively. By contrast, differences in the concentrations of reducing sugars between materials were non-significant. However, the reducing sugars-to-sucrose ratio of non-grafted material registered consistently higher nominal values in both years, although the high experimental error rendered these differences statistically non-significant. Malic acid was the dominant organic acid in the carob flour of both grafted and non-grafted material, accounting for 74.7% and 79.1% of total acids content, respectively. However, the concentrations of malic, citric, and oxalic acid as well as total acids did not differ significantly between grafted and non-grafted material. Similarly, protein content did not differ significantly in carob flour milled from grafted (4.69% *w*/*w*) and non-grafted material (4.85% *w*/*w*).

### 3.5. Chromatometric Correlations with Carob Flour Compositional Variables

**Figure 2:**
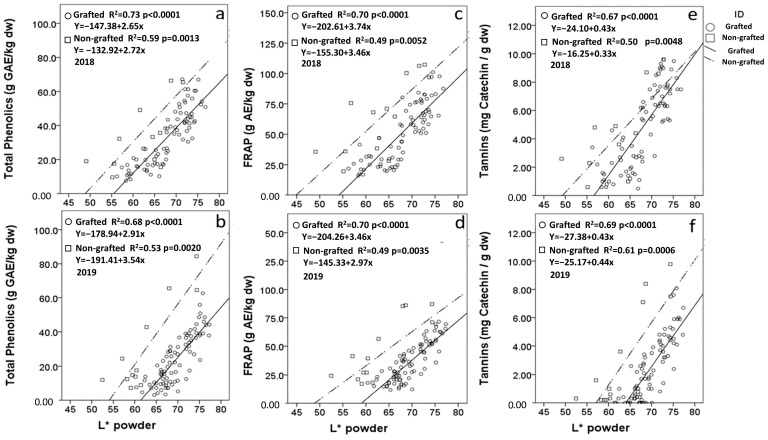
Pearson correlation of total phenolics (**a**,**b**), FRAP (**c**,**d**), and tannins (**e**,**f**) with flour lightness (L*) of non-grafted and grafted source material harvested in two constitutive years (2018 and 2019).

Overall, the correlation of chromatometric parameter L* (lightness) with the compositional attributes of carob flour was similar for grafted and non-grafted material (Table 3). The in vitro antioxidant capacity of carob flour correlated positively with L*, especially for the FRAP assay (*r* = 0.838–0.836; *p* > 0.01). A significant positive correlation was also observed between L* and total phenolics and tannins contents, which denoted that carob flours of lighter color tend to have higher antioxidant content and capacity. A positive correlation with L* was also observed for sucrose and malic acid contents, whereas the concentration of reducing sugars and their ratio to sucrose yielded significant negative correlations. The total sugars concentration of carob flour demonstrated low correlation coefficients within the distinctive populations of grafted and non-grafted samples. However, the correlation was significant in both years of the study when examined for the entire sample population. This likely reflects the impact on the correlation with L* of the lower sugar content and darker flours obtained from the non-grafted samples. Apart from the above compositional variables, the lightness-L* of carob flour was also found to correlate significantly with the elevation (altitude) of the trees sampled for both grafted and non-grafted material (Table 3). This is an observation of great significance for the carob industry as it suggests that carob flours milled from pods collected from higher altitudes tend to be lighter in color and, by correlation, higher in phenolics, tannins, sucrose, and malic acid concentrations, as well as in antioxidant capacity (Figure 2). These flours also tend to have a lower relative content of reducing sugars fructose and glucose, the possible role of which in the darkening of carob pulp and, consequently, carob flour warrants further investigation. Conversely, pods sourced from lower altitudes tend to have higher levels of reducing sugars and yield darker flours, possibly because their accelerated ripening process does not facilitate the conversion of reducing sugars to sucrose. This differentiation of source material based on relative content in reducing sugars might be especially critical when prior to milling, carob kibbles are roasted at temperatures conducive to Maillard reactions [34,35].

The oxidation of polyphenols by polyphenol oxidase (PPO) and peroxidase (POX) is most probably implicated in the darker flour (lower L*) milled from carobs of certain geographical and altitudinal sources. It is widely known that the activity of these ubiquitous enzymes reduces the quality of horticultural products and results in the loss of color, flavor, and the production of orthoquinones that form dark-colored melanin pigments that polymerize non-enzymatically [36]. Stress conditions of heat, light, and moisture to which polyphenols are highly sensitive tend to aggravate the oxidation of polyphenols to orthoquinones. Such conditions of stress (e.g., higher temperature and relative humidity) are more prevalent at low altitudes, especially in coastal areas, which might contribute to the darker flour obtained from carobs sourced from these areas. The correlation coefficients linking altitude to flour color and key compositional variables are generally high; however, it is worth noting that it could have been even higher in the absence of outlying samples from coastal microclimates that register lower winter temperatures and have igneous soils of high drainage capacity. It should also be taken into consideration that the inclusion of non-grafted material in the sample population lowered the correlation coefficients of carob flour L* with phenolics, tannins, and antioxidant capacity; therefore, it compromised the prognostic value of flour color for compositional variables linked to sensory and functional quality.

**Figure 3:**
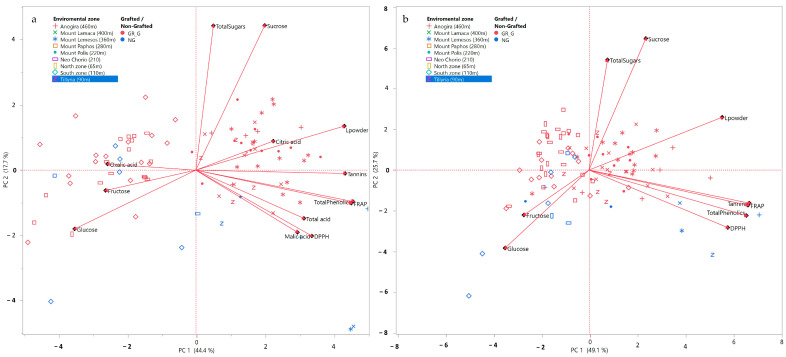
PCA analysis of carob flour compositional and qualitative traits (L*, fructose, glucose, sucrose, total sugars, DPPH, FRAP, total phenolics, tannins, organic acids) for the entire sampled population, including genotype, sampling area, and corresponding altitudinal range, for two consecutive years 2018 (**a**) and 2019 (**b**).

The above findings are reinforced by the PCA analysis performed on all carob flour compositional and qualitative traits for the entire sampled population, including genotype and sampling area with corresponding altitudinal range, for two consecutive years (Figure 3). The first two principal components of the PCA analysis explained 62.1% and 74.8% of the variability in 2018 and 2019, respectively. In both years, PC 1 positively correlated with tannins, total phenolics, and antioxidant activity (DPPH and FRAP), which constituted strongly correlated variable traits. Carob flour L* also consistently and positively correlated with PC1, while glucose and fructose correlated negatively. Sucrose and total sugars positively correlated with PC2. The results of the PCA analysis confirm that carob flours of darker color (lower L*) are characterized by higher relative content of reducing sugars.

In both years, trees grown in the areas of Anogira, Mountainous Polis, Limassol, Larnaca, and Tillyria were plotted on the positive side of PC1, whereas trees grown in the north and south zones, Neo Chorio, and Mountainous Pafos were plotted on the negative side. In general, trees plotted on the positive side of PC1 originated from areas of high altitude and long distances from the sea, with the exception of Tillyria (low altitude, short distance from the sea). Finally, PC2 discriminated between grafted from non-grafted trees.

### 3.6. Volatile Organic Compounds (VOCs)

A total of 44 different volatile constituents were detected in carob flour obtained from grafted and non-grafted material, which amounted to 97–99% of the total volatile composition (Table 4 and Table 5). The VOCs presently identified in carob powder comprised five principal groups: acids, esters, aldehydes, ketones, and alcohols. The same categories of VOCs were reported in past studies investigating the aromatic profile of mature carobs sourced from different countries [30,37]. Acids comprised the most abundant group of VOCs identified, which accounted for 68.42% and 68.86% of the total VOCs identified in flour from grafted and non-grafted carobs, respectively. The most abundant volatile compound present in carob flour was 2-methyl-propanoic acid (isobutyric acid), with a relative abundance of 45.98% and 47.33% in flour from grafted and non-grafted carobs, respectively. Isobutyric acid is responsible for the cheesy acidic buttery aroma widely considered the aroma signature of the carob pod [11,29,37]. Isobutyric acid is also a key component of the cocoa powder volatilome, which renders carob powder an attractive substitute for carob powder [38]. Esters comprised the second most abundant category of VOCs, with a total relative abundance of 26.81% and 24.99% for grafted and non-grafted materials, respectively. It is worth noting that the major esters identified were the esterification products of the three major volatile acids also present in carob flour (isobutyric, butanoic, and hexanoic). The remaining VOC categories in declining relative abundance were ketones, alcohols, and aldehydes. Differences between grafted and non-grafted material with respect to the relative abundance of the five VOCs categories were non-significant, and the same was true for all key compounds identified. It may therefore be inferred that the flours produced from grafted and non-grafted carobs have very similar aroma profiles reflecting very similar volatile compounds composition. Unsurprisingly, the correlations between individual and categorical VOCs and flour colorimetry were also very similar for grafted and non-grafted material. All individual organic acids correlated negatively with L*; however, individual compounds within the other VOC categories presented variable trends in their correlation with L*. Isobutyric acid, which was the most abundant of the VOCs identified, did not correlate significantly with L*. Interestingly, butanoic and propanoic acids, which contribute unpleasant aromas noted as sharp acetic (product of anaerobic fermentation) and pungent body odor, respectively, did not correlate significantly with flour L*. The acid yielding the highest negative correlation with L* (r = −0.723; *p* < 0.01) was isovaleric acid responsible for the fruity sweet apple pineapple aroma, denoting an increased relative abundance of this aroma quality in darker carob flours. Overall, it might be concluded that the relative abundance of organic acids responsible for less pleasant aromas is higher in darker carob flours.

The methyl and ethyl esters of the dominant isobutyric acid, responsible, respectively, for fruity green apple grape peach aroma and sweet fruity ethereal rummy aroma [39], registered significant positive correlations with L* (r = 0.747 and r = 0.659; *p* < 0.01), denoting increasing abundance in lighter-colored flours. The methyl esters of butanoic, acetic, and pentanoic acids, which generally contributed to ethereal sweet fruity aromas, presented significant positive correlations with L*, denoting increasing relative abundance in lighter-colored flours. By contrast, some of the less abundant esters, such as octanoic acid methyl ester, propanoic acid, 2-methyl-, 2-methylbutyl ester, and the 2-methylpropyl esters of hexanoic and butanoic acids correlated negatively with L*. Alcohols were generally more abundant in darker flours (negative correlation with L*); however, only 2-methyl-1-propanol (ethereal winey) correlated significantly with L* (r = −0.732; *p* < 0.01). Of ketones, only 2-pentanone (sweet fruity ethereal woody) correlated significantly with L* (r = 0.659; *p* < 0.01). Aldehydes were also generally more abundant in darker flours (negative correlation with L*); however, only benzaldehyde demonstrated a significant correlation with L*. According to a previous study, aldehydes and ketones were also found in higher abundance in roasted (darker-colored) carob flour compared with unroasted flour [29].

Overall, it might be concluded from the analysis of VOCs composition in unroasted carob flour that grafted and non-grafted carobs demonstrated very similar aroma profiles. It is also evident that lighter-colored flours (higher L*) demonstrate a VOCs composition lower in acids, aldehydes, and alcohols but richer in esters and, to a lesser extent, ketones. It might therefore be inferred that the more pleasant aromas (sweet ethereal fruity) produced mainly by esters and ketones are more abundant in lighter-colored carob flours, whereas darker flours tend to be richer in acids responsible for less pleasant and more intense aromas.

**Table 4:** Means and standard error (Std Error) of the relative abundance of VOCs found in carob flour milled from grafted (GR) and non-grafted (NGR) source material and significance of t-test for differences in VOCs between grafted and non-grafted material. Pearson correlation coefficients and significance between flour lightness (L*) with VOCs overall genotypes. The VOCs list includes chemical formula, PubChem CID, and odor description.

Volatile Compound Name	Molecular Formula	PubChem CID	Odor Description †	Mean ± Std Error (GR)	Mean ± Std Error (NGR)	*t*-Test	Correlation L (Overall)
***ACIDS***							
Propanoic acid, 2-methyl-(isobutyric acid)	C4H8O2	6590	acidic, sour, cheesy, dairy buttery, rancid	45.98 ± 1.05 ***	47.33 ± 3.29 ***	ns	−0.021
Hexanoic acid (caproic acid)	C6H12O2	8892	sour, fatty, sweaty, cheesy	7.40 ± 0.48 ***	8.23 ± 0.94 ***	ns	−0.529 *
Butanoic acid (butyric acid)	C4H8O2	264	sharp, acetic, cheesy, buttery, fruity (product of anaerobic fermentation)	7.37 ± 0.29 ***	5.99 ± 0.73 ***	ns	−0.256
Acetic acid	C2H4O2	176	sour	5.09 ± 0.39 ***	4.47 ± 0.64 ***	ns	−0.321
Butanoic acid, 3-methyl-(isovaleric acid)	C5H10O2	10430	fruity, sweet, apple, pineapple, tutti, frutti	2.23 ± 0.16 ***	2.62 ± 0.19 ***	ns	−0.723 **
Butanoic acid, 2-methyl-(2-methylbutyric acid)	C5H10O2	8314	pungent, acidic, cheesy, roquefort cheese,	0.18 ± 0.03 **	0.07 ± 0.03 ***	*	−0.184
Propanoic acid (propionic acid)	C3H6O2	1032	pungent and unpleasant smell somewhat resembling body odor	0.13 ± 0.001 **	0.11 ± 0.04 ***	ns	−0.063
Pentanoic acid (valeric acid)	C5H10O2	7991	pcidic, sharp, cheesy, sour, milky, tobacco, fruity	0.04 ± 0.01 ***	0.03 ± 0.02 ***	ns	−0.081
**Total Acids**				68.42 ± 1.61 ***	68.86 ± 2.77 ***	**ns**	−0.588 *
***ESTERS***							
Hexanoic acid, methyl ester	C7H14O2	7824	sweet, fruity, pineapple, waxy, green, banana	8.09 ± 0.54 ***	7.92 ± 1.25 ***	ns	0.300
Propanoic acid, 2-methyl-, methyl ester (methyl isobutyrate)	C5H10O2	7749	fruity, green apple, pear, tart, grape berry, ripe berry, winey, peach	6.50 ± 0.61 ***	5.97 ± 1.10 ***	ns	0.747 **
Butanoic acid, methyl ester	C5H10O2	12180	fruity, apple, sweet, banana, pineapple	6.22 ± 0.38 ***	4.64 ± 0.88 ***	ns	0.643 **
Acetic acid, methyl ester	C3H6O2	6584	ethereal, sweet, fruity	2.93 ± 0.12 **	2.48 ± 0.21 **	ns	0.679 **
Octanoic acid, methyl ester	C9H18O2	8091	waxy, green, sweet, orange, aldehydic, vegetable, herbal	0.66 ± 0.05 ***	0.92 ± 0.13 ***	*	−0.686 **
Pentanoic acid, methyl ester (methyl valerate)	C6H12O2	12206	sweet, green, fruity, apple, pineapple, nutty	0.38 ± 0.01 ***	0.28 ± 0.07 ***	ns	0.659 **
Propanoic acid, 2-methyl-, 3-methylbutyl ester (isoamyl isobutyrate)	C9H18O2	519786	ethereal, fruity, fruit tropical, fruit pineapple, grape, skin banana	0.33 ± 0.08 ***	0.23 ± 0.12 ***	ns	0.028
Propanoic acid, 2-methyl-, ethyl ester (isobutyric ethyl ester)	C6H12O2	7342	sweet, fruity, ethereal, rummy	0.29 ± 0.05 ***	0.22 ± 0.06 ***	ns	0.633 **
Butanoic acid, 2-methyl-, methyl ester	C6H12O2	13357	ethereal, estery, fruity, tutti, frutti apple, green apple, lily of the valley, powdery, fatty	0.28 ± 0.03 ***	0.29 ± 0.06 **	ns	0.561 *
Propanoic acid, 2-methyl-, 2-methylpropyl ester	C8H16O2	7351	waxy, green, sweet, orange, and aldehydic with vegetative and herbal nuances	0.20 ± 0.11 ***	0.37 ± 0.15 ***	ns	−0.253
Propanoic acid, methyl ester (methyl propionate )	C4H8O2	11124	fresh, rummy, fruity, strawberry, apple	0.17 ± 0.01 ***	0.12 ± 0.01 ***	ns	0.605 *
Butanoic acid, ethyl ester	C6H12O2	7762	fruity, green apricot, pear, banana	0.15 ± 0.02 ***	0.08 ± 0.02 ***	ns	0.437
Hexanoic acid, ethyl ester	C8H16O2	31265	sweet, fruity, pineapple, green peach, tropical	0.15 ± 0.02 ***	0.14 ± 0.04 ***	ns	0.058
Butanoic acid, 3-methylbutyl ester	C9H18O2	7795	fruity, green apricot, pear, banana	0.13 ± 0.07 ***	0.61 ± 0.25 ***	ns	−0.383
Propanoic acid, 2-methyl-, 2-methylbutyl ester	C9H18O2	97883	fruity, ethereal, tropical, banana	0.12 ± 0.03 **	0.24 ± 0.13 ***	ns	−0.647 **
Ethyl acetate	C4H8O2	8857	ethereal, fruity, sweet, weedy, green/ester	0.09 ± 0.02 ***	0.05 ± 0.02 ***	ns	0.362
Propanoic acid, 2-methyl-, hexyl ester (isobutyric hexyl ester)	C10H20O2	16872	sweet, ethereal, fruity, alcoholic fusel rummy	0.07 ± 0.02 ***	0.15 ± 0.06 ***	ns	−0.386
Hexanoic acid, 2-methylpropyl ester	C10H20O2	7775	fruity, apple, sweet, banana, pineapple	0.04 ± 0.03 ***	0.22 ± 0.15 ***	ns	−0.821 **
Butanoic acid, 2-methylpropyl ester	C8H16O2	10885	sweet, fruity, pineapple, tutti, frutti, rum, cherry, apple, overripe fruit, bubble gum	0.02 ± 0.02 ***	0.05 ± 0.04 ***	ns	−0.589 *
**Total Esters**				26.81 ± 1.49 ***	24.99 ± 2.93 ***	**ns**	0.550 *
***ALCOHOLS***			fusel, alcoholic, whiskey, fruity, banana sweet, green, fruity, apple, pineapple, nutty ethereal, winey mild, ethereal odor ^a^ waxy, green, creamy, citrus, orange, cheesy fruity				
2-Heptanol	C7H16O	10976		0.20 ± 0.02 ***	0.28 ± 0.05 ***	ns	−0.059
1-Butanol, 3-methyl-(isoamyl alcochol)	C5H12O	31260		0.08 ± 0.02 ***	0.11 ± 0.04 ***	ns	−0.207
1-Propanol, 2-methyl-	C4H10O	6560		0.02 ± 0.01 ***	0.06 ± 0.04 ***	ns	−0.732 **
2-Propanol, 1-methoxy-	C4H10O2	7900		0.008 ± 0.01 ***	ND	ns	−0.140
2-Nonanol	C9H20O	12367		0.02 ± 0.01 ***	0.07 ± 0.02 ***	ns	−0.123
**Total Alcohols**				0.32 ± 0.05 ***	0.51 ± 0.11 ***	**ns**	**−0.355**
***KETONES***			fruity, spicy, sweet, herbal, coconut, woody fresh, sweet, green, weedy, earthy, herbal sweet, fruity, ethereal, winey, banana, woody pungent, sweet, creamy, buttery alcoholic, musty, woody buttery, sweet, creamy, pungent, caramellic				
2-Heptanone	C7H14O	8051		2.24 ± 0.21 ***	3.19 ± 0.60 ***	ns	0.125
2-Nonanone	C9H18O	13187		0.62 ± 0.05 ***	0.82 ± 0.15 ***	ns	−0.269
2-Pentanone	C5H10O	7895		0.44 ± 0.08 ***	0.46 ± 0.19 ***	ns	0.659 **
Acetoin	C4H8O2	179		0.41 ± 0.05 ***	0.28 ± 0.04 **	ns	−0.238
Acetone	C3H6O	180		0.26 ± 0.03 ***	0.27 ± 0.06 **	ns	−0.012
2,3-Butanedione	C4H6O2	650		0.20 ± 0.02 ***	0.17 ± 0.04 ***	ns	−0.385
**Total Ketones**				4.17 ± 0.31 ***	5.18 ± 0.92 ***	**ns**	**0.156**
***ALDEHYDES***			ethereal, aldehydic, chocolate, peach, fatty strong, sharp, sweet, bitter almond, cherry fresh, aldehydic, floral, pungent pungent, ethereal, aldehydic, fruity characteristic aromatic odor ^a^				
Butanal, 3-methyl-	C5H10O	11552		0.08 ± 0.01 ***	0.08 ± 0.01 **	ns	−0.192
Benzaldehyde	C7H6O	240		0.03 ± 0.02 ***	0.13 ± 0.09 ***	ns	−0.686 **
Propanal, 2-methyl-(isobutyraldehyde)	C4H8O	6561		0.02 ± 0.01 ***	0.04 ± 0.01 ***	ns	−0.200
Acetaldehyde	C2H4O	177		0.01 ± 0.01 ***	0.01 ± 0.01 ***	ns	−0.017
**Total Aldehyde**				0.15 ± 0.02 ***	0.27 ± 0.07 ***	**ns**	−0.758 **
**Toluene**	C7H8	1140		0.14 ± 0.04 ***	0.19 ± 0.06 ***	ns	−0.257

ns = non-significant, * *p* > 0.05, ** *p* > 0.01, *** *p* > 0.0001, ND = none detected. † Odor description was adopted from the Good Scents Company 2021 (source: McGorrin 2021 [40]), apart from ^a^ in which odor description comes from PubChem (https://pubchem.ncbi.nlm.nih.gov/ (accessed on 10 January 2023)).

**Table 5:** Stepwise regression analysis of traits correlating highly to flour lightness (L*) for grafted (GR) and non-grafted (NGR) source materials in 2018 and 2019.

Year	Category	Variable Enter	Standardized Beta	R^2^	R^2^ Change	F Change	Prop.
2018	GR	Total Phenolics (HCL)	0.685	0.734	0.734	207.06	≥0.001
		Glucose	−0.325	0.810	0.076	29.65	≥0.001
	NGR	Total Phenolics (HCL)	0.547	0.592	0.592	17.39	0.0013
		Glucose	−0.424	0.722	0.130	5.14	0.0400
2019	GR	Total Phenolics (HCL)	0.791	0.681	0.681	168.54	≥0.001
		Sucrose	0.205	0.742	0.061	18.62	≥0.001
		Fructose	−0.154	0.764	0.022	7.05	0.0090
	NGR	Total Phenolics (HCL)	0.606	0.534	0.534	14.91	0.0019
		Sucrose	0.458	0.728	0.194	8.58	0.0100

### 3.7. Regression Analysis of Carob Flour L* and Compositional Variables

Stepwise regression analysis indicated that in 2018 the total phenolics and glucose concentrations accounted for 73.4% and 7.6% of the variability in the flour chromatic variable L* for the grafted material (R2 change; Table 4). Corresponding values for non-grafted material in the same year were 59.2% and 13.0%. In 2019, the total phenolics concentration remained the most influential compositional variable explaining variability in flour L*, accounting for 68.1% and 53.4% of the variability in L* for grafted and non-grafted material. In the same year, sucrose and fructose contents explained 6.1% and 2.2% of the variability in L* for grafted material, whereas in the case of non-grafted material, sucrose was the key influential sugar component, accounting for 19.4% of the variability in L*. The standardized beta values were positive for phenolics and sucrose and negative for reducing sugars.

Regression analysis for 2018 and 2019 revealed significant linear correlations for total phenolics content, FRAP antioxidant capacity, and tannins content with carob flour L* colorimetry (Figure 2). For most of the linear regressions of the above variables with L*, the slopes for grafted and non-grafted material were either unequal or equal but with unequal intercepts with the *x*-axis. The intercepts of regression lines with the L* *x*-axis were, in all cases, lower for the non-grafted material. It is, therefore, evident that carob flours obtained from non-grafted material yielded higher values of phenolics, FRAP, and tannins compared to grafted material of the same L* value. Practically, this means that darker-colored flour from non-grafted material may have the same values for these compositional and functional variables as lighter-colored flour from grafted material. Shifting of the L* *x*-axis intercept was also observed between the two sampling years for both types of material sampled. This finding implies that although a strong linear regression may define the correlation of L* with key compositional variables, the exact prognostication value of L* for these compositional variables varies from year to year. It may be therefore inferred that the L* value of carob flour is a reliable index for ranking same-year carob flour samples for key compositional variables; however, to establish a reliable predictive model of compositional values across years, it is necessary to accumulate compositional data and L* values from multiple years of harvest.

### 3.8. PCA Analysis of Compositional Variables and Sample Origins

The first two components of the PCA analysis explained 62.1% and 74.8% of the variability in 2018 and 2019, respectively (Figure 3). In both years, PC 1 positively correlated with tannins, total phenolics, and antioxidant activity (DPPH and FRAP). These variables were strongly related. Carob flour L* also consistently and positively correlated with PC1, while glucose and fructose correlated negatively. Sucrose and total sugars positively correlated with PC2. The results of the PCA analysis confirm that carob flours of darker color (lower L*) are characterized by higher relative content of reducing sugars.

In both years, trees grown in the areas of Anogira, Mountainous Polis, Limassol, Larnaca, and Tillyria were plotted to the positive side of PC1, whereas trees grown in north and south zones, Neo Chorio, and Mountainous Pafos were plotted to the negative side. In general, trees plotted to the positive side of PC1 originated from areas of high altitude and long distance from the sea, with the exception of Tillyria (low altitude, short distance from the sea). PC2 discriminated between grafted from non-grafted trees.

## 4. Conclusions

Chromatic lightness (L*) was higher and more stable across seasons in carob flour milled from grafted than from genetically variant non-grafted source material. Grafted material yielded carob flour of higher L* and total sugars concentrations, whereas non-grafted material had a higher antioxidant capacity and total phenolic compounds across seasons, while tannins concentrations were similar for both types of the source material. Flours milled from grafted and non-grafted carobs shared similar volatile compositions. Nonetheless, lighter-colored (higher L*) flours were lower in acids, aldehydes, and alcohols responsible for less pleasant aromas but richer in esters and ketones that impart pleasant, sweet ethereal fruity aromas. Overall, the correlations of carob flour L* with compositional attributes were similar in pattern for grafted and non-grafted material, but the prognostication potential of L* was more robust for grafted material owing putatively to its narrower genetic basis. The antioxidant capacity, the total phenolic, tannins, and sucrose concentrations correlated positively with L*, which denoted increased levels in lighter carob flours. Such flours were milled from pods collected at higher altitudes. These flours also tended to have a lower relative content of reducing sugars fructose and glucose, the possible role of which in the darkening of carob pulp and flour warrants further investigation. Although a strong linear regression generally defines the correlation of L* with key carob flour compositional variables, the exact prognostication of these variables based on L* may vary from year to year. It may be therefore inferred that carob flour L* is a particularly reliable index for ranking same-year carob flours for key compositional attributes that may be further improved in resilience by drawing on multiple-year data.

## Figures and Tables

**Table 1:** Means, standard error (Std. Error), maximum, and minimum values for L* and compositional traits of grafted and non-grafted source material for the 2018 and 2019 harvest seasons.

	Grafted (*n* = 68)				Non-Grafted (*n* = 13)				*t*-Test
Trait	Mean	Std. Error	Minimum	Maximum	Mean	Std. Error	Minimum	Maximum	Significance
L* powder (2018)	68.05	0.61	56.24	76.78	63.54	1.95	49.10	73.54	**
L* powder (2019)	69.75	0.46	60.37	77.33	63.38	1.63	52.47	74.40	**
DPPH (g AAE/kg dw) (2018)	9.23	0.39	3.19	20.87	14.26	1.45	5.38	24.14	**
DPPH (g AAE/kg dw) (2019)	7.18	0.49	0.88	30.72	13.17	2.84	1.73	39.04	ns
FRAP (g AAE/kg dw) (2018)	51.90	2.72	15.98	101.20	64.61	9.60	19.57	130.48	ns
FRAP (g AAE/kg dw) (2019)	37.04	1.89	11.88	71.63	43.14	6.90	17.19	86.98	ns
Total Phenolics (HCL) (g GAE/kg dw) (2018)	33.39	1.89	7.55	67.17	39.99	6.88	9.57	85.35	ns
Total Phenolics (HCL) (g GAE/kg dw) (2019)	24.36	1.61	3.02	62.67	32.92	7.90	7.30	99.18	ns
Tannins (mg Catechin/g dw) (2018)	4.87	0.32	0.50	9.59	4.70	0.91	0.60	10.79	ns
Tannins (mg Catechin/g dw) (2019)	2.46	0.23	0.00	8.08	2.75	0.92	0.00	9.78	ns
Total Phenolics-Acetate (g GAE/kg dw) (2018)	10.53	0.34	5.23	18.51	15.07	1.85	7.75	29.05	*
Fructose (g/100 g dw) (2018)	10.14	0.13	8.05	13.92	9.18	0.39	6.64	11.68	**
Fructose (g/100 g dw) (2019)	7.56	0.11	5.20	10.64	7.02	0.56	4.59	12.02	ns
Glucose (g/100 g dw) (2018)	5.16	0.13	3.14	9.52	5.45	0.41	2.37	8.54	ns
Glucose (g/100 g dw) (2019)	3.46	0.09	1.67	6.09	4.02	0.52	1.49	9.27	ns
Sucrose (g/100 g dw) (2018)	35.34	0.44	23.20	43.38	29.25	1.73	12.10	35.18	**
Sucrose (g/100 g dw) (2019)	30.99	0.39	23.29	38.05	24.96	1.33	12.58	32.67	***
Total Sugars (g/100 g dw) (2018)	50.64	0.32	44.33	57.01	43.88	1.52	27.85	50.94	**
Total Sugars (g/100 g dw) (2019)	42.01	0.37	34.45	49.19	35.99	0.79	30.46	41.19	***
F/S (2018)	0.29	0.01	0.21	0.57	0.35	0.05	0.19	0.87	ns
F/S (2019)	0.25	0.01	0.16	0.44	0.32	0.05	0.15	0.96	ns
G/S (2018)	0.15	0.01	0.08	0.41	0.21	0.03	0.07	0.43	ns
G/S (2019)	0.11	0.00	0.05	0.26	0.19	0.05	0.05	0.74	ns
(F + G)/S (2018)	0.44	0.01	0.30	0.98	0.55	0.07	0.26	1.30	ns
(F + G)/S (2019)	0.36	0.01	0.22	0.70	0.51	0.10	0.20	1.69	ns
Malic acid (mg/g dw) (2018)	5.81	0.17	1.49	9.01	6.52	0.59	3.99	10.71	ns
Citric acid (mg/g dw) (2018)	1.66	0.05	0.58	2.90	1.43	0.13	0.40	2.52	ns
Oxalic acid (mg/g dw) (2018)	0.31	0.01	0.21	0.44	0.30	0.01	0.22	0.39	ns
Total acid (mg/g dw) (2018)	7.78	0.19	2.57	11.25	8.25	0.64	5.45	13.45	ns
Protein % (*w*/*w*) (2018)	4.68	0.09	2.87	7.44	4.85	0.36	2.18	7.87	ns

ns = non-significant, * *p* > 0.05, ** *p* > 0.01, *** *p* > 0.0001.

**Table 2:** Pearson correlation coefficients (r), significance of correlations (Sig), and significance of t-test for correlations between years for grafted and non-grafted and all genotypes (overall).

	Grafted (*n* = 68)			Non-Grafted (*n* = 13)			Overall (*n* = 81)		
Trait	Correlation	Sig.	*t*-Test	Correlation	Sig.	*t*-Test	Correlation	Sig.	*t*-Test
L* powder	0.638	***	*	0.694	**	ns	0.693	***	*
DPPH	0.539	***	***	0.771	**	ns	0.685	***	**
FRAP	0.734	***	***	0.594	*	*	0.699	***	***
Total Phenolics	0.734	***	***	0.550	ns	ns	0.663	***	***
Tannins	0.663	***	***	0.501	ns	ns	0.612	***	***
Fructose	0.311	**	***	0.501	ns	**	0.389	**	***
Glucose	0.303	*	***	0.517	ns	*	0.383	**	***
Sucrose	0.369	**	***	0.712	**	**	0.586	***	***
Total Sugars	0.320	**	***	0.376	ns	**	0.517	***	***
F/S	0.337	**	***	0.662	*	ns	0.575	***	**
G/S	0.309	*	***	0.817	**	ns	0.621	***	***
(F + G)/S	0.333	**	***	0.732	**	ns	0.608	***	***

ns = non-significant, * *p* > 0.05, ** *p* > 0.01, *** *p* > 0.0001. F = fructose, G = glucose, S = sucrose.

**Table 3:** Pearson correlation coefficients (corr) and correlation significance of flour lightness (L*) with compositional traits and elevation for grafted (GR, *n* = 68), non-grafted (NGR, *n* = 13), and all genotypes (overall, *n* = 81) in 2018 and 2019.

Trait	L* Corr (GR)	L* Corr (NGR)	L* Corr (Overall)
DPPH (2018)	0.624 **	0.450	0.369 **
DPPH (2019)	0.666 **	0.639 *	0.340 **
FRAP (2018)	0.838 **	0.701 **	0.696 **
FRAP (2019)	0.836 **	0.703 **	0.608 **
Total Phenolics (2018)	0.857 **	0.769 **	0.748 **
Total Phenolics (2019)	0.825 **	0.731 **	0.567 **
Tannins (2018)	0.820 **	0.705 **	0.764 **
Tannins (2019)	0.833 **	0.783 **	0.669 **
Fructose (2018)	−0.520 **	−0.387	−0.369 **
Fructose (2019)	−0.284 *	−0.496	−0.243 *
Glucose (2018)	−0.688 **	−0.711 **	−0.660 **
Glucose (2019)	−0.329 **	−0.643 **	−0.452 **
Sucrose (2018)	0.607 **	0.169	0.537 **
Sucrose (2019)	0.331 **	0.623 *	0.557 **
Total Sugars (2018)	0.329 **	−0.101	0.306 **
Total Sugars (2019)	0.182	0.274	0.419 **
F/S (2018)	−0.632 **	−0.236	−0.492 **
F/S (2019)	−0.392 **	−0.576 *	−0.496 **
G/S (2018)	−0.679 **	−0.512	−0.645 **
G/S (2019)	−0.396 **	−0.616 *	−0.525 **
(F + G)/S (2018)	−0.664 **	−0.347	−0.571 **
(F + G)/S (2019)	−0.407 **	−0.596 *	−0.516 **
Malic acid (2018)	0.457 **	0.586 *	0.440 **
Citric acid (2018)	0.390 **	0.442	0.419 **
Oxalic acid (2018)	−0.524 **	−0.091	−0.397 **
Total acid (2018)	0.488 **	0.624 *	0.491 **
Protein (2018)	−0.192	0.051	−0.117
Altitude (2018)	0.521 **	0.673 **	0.528 **
Altitude (2019)	0.547 **	0.608 *	0.472 **

F = fructose, G = glucose, S = sucrose. * Correlation is significant at the 0.05 level (2-tailed). ** Correlation is significant at the 0.01 level (2-tailed).

## Data Availability

The raw data supporting the conclusions of this article will be made available by the authors, without undue reservation.

## References

[1] Batlle I., Tous J. (1997). Carob tree: *Ceratonia siliqua* L.-Promoting the conservation and use of underutilized and neglected crops. Bioversity Int..

[2] Kyratzis A.C., Antoniou C., Papayiannis L.C., Graziani G., Rouphael Y., Kyriacou M.C. (2021). Pod Morphology, Primary and Secondary Metabolite Profiles in Non-grafted and Grafted Carob Germplasm Are Configured by Agro-Environmental Zone, Genotype, and Growing Season. Front. Plant Sci..

[3] Tous J., Romero A., Batlle I. (2013). The Carob tree: Botany, horticulture, and genetic resources. Horticultural Reviews.

[4] Bouzouita N., Khaldi A., Zgoulli S., Chebil L., Chekki R., Chaabouni M., Thonart P. (2007). The analysis of crude and purified locust bean gum: A comparison of samples from different carob tree populations in Tunisia. Food Chem..

[5] Avallone R., Plessi M., Baraldi M., Monzani A. (1997). Determination of Chemical Composition of Carob (*Ceratonia siliqua*): Protein, Fat, Carbohydrates, and Tannins. J. Food Compos. Anal..

[6] Kyriacou M.C., Antoniou C., Rouphael Y., Graziani G., Kyratzis A. (2021). Mapping the Primary and Secondary Metabolomes of Carob (*Ceratonia siliqua* L.) Fruit and Its Postharvest Antioxidant Potential at Critical Stages of Ripening. Antioxidants.

[7] Čepo D.V., Mornar A., Nigović B., Kremer D., Radanović D., Vedrina Dragojević I. (2014). Optimization of roasting conditions as an useful approach for increasing antioxidant activity of carob powder. LWT—Food Sci. Technol..

[8] Craig W.J., Nguyen T.T. (1984). Caffeine and Theobromine Levels in Cocoa and Carob Products. J. Food Sci..

[9] Benković M., Belščak-Cvitanović A., Bauman I., Komes D., Srečec S. (2017). Flow properties and chemical composition of carob (*Ceratonia siliqua* L.) flours as related to particle size and seed presence. Food Res. Int..

[10] Sęczyk Ł., Świeca M., Gawlik-Dziki U. (2016). Effect of carob (*Ceratonia siliqua* L.) flour on the antioxidant potential, nutritional quality, and sensory characteristics of fortified durum wheat pasta. Food Chem..

[11] Antoniou C., Kyratzis A.C., Soteriou G.A., Rouphael Y., Kyriacou M.C. (2021). Configuration of the Volatile Aromatic Profile of Carob Powder Milled From Pods of Genetic Variants Harvested at Progressive Stages of Ripening from High and Low Altitudes. Front. Nutr..

[12] Yousif A.K., Alghzawi H.M. (2000). Processing and characterization of carob powder. Food Chem..

[13] Şahin H., Topuz A., Pischetsrieder M., Özdemir F. (2009). Effect of roasting process on phenolic, antioxidant and browning properties of carob powder. Eur. Food Res. Technol..

[14] Eldeeb G.S.S., Mosilhey S.H. (2022). Roasting temperature impact on bioactive compounds and PAHs in Carob powder (*Ceratonia siliqua* L.). J. Food Sci. Technol..

[15] Berna A., Pérez-Gago M.B., Guardiola V.G., Salazar D., Mulet A. (1997). Effect of Temperature on Isobutyric Acid Loss during Roasting of Carob Kibble. J. Agric. Food Chem..

[16] Kumazawa S., Taniguchi M., Suzuki Y., Shimura M., Kwon M.-S., Nakayama T. (2002). Antioxidant Activity of Polyphenols in Carob Pods. J. Agric. Food Chem..

[17] Fellows P.J. (2009). Food Processing Technology: Principles and Practice.

[18] Kyriacou M.C., Rouphael Y. (2018). Towards a new definition of quality for fresh fruits and vegetables. Sci. Hortic..

[19] Ioannou G.D., Savva I.K., Christou A., Stavrou I.J., Kapnissi-Christodoulou C.P. (2023). Phenolic Profile, Antioxidant Activity, and Chemometric Classification of Carob Pulp and Products. Molecules.

[20] Nguyen Q.T., Nguyen T.T., Le V.N., Nguyen N.T., Truong N.M., Hoang M.T., Pham T.P., Bui Q.M. (2023). Towards a Standardized Approach for the Geographical Traceability of Plant Foods Using Inductively Coupled Plasma Mass Spectrometry (ICP-MS) and Principal Component Analysis (PCA). Foods.

[21] McGuire R.G.J.H. (1992). Reporting of objective color measurements. HortScience.

[22] Singleton V.L., Orthofer R., Lamuela-Raventós R.M. (1999). Analysis of total phenols and other oxidation substrates and antioxidants by means of folin-ciocalteu reagent. Methods in Enzymology.

[23] Sun B., Ricardo-da-Silva J.M., Spranger I. (1998). Critical Factors of Vanillin Assay for Catechins and Proanthocyanidins. J. Agric. Food Chem..

[24] Sepperer T., Hernandez-Ramos F., Labidi J., Oostingh G.J., Bogner B., Petutschnigg A., Tondi G. (2019). Purification of industrial tannin extract through simple solid-liquid extractions. Ind. Crops Prod..

[25] Antoniou C., Kyratzis A., Rouphael Y., Stylianou S., Kyriacou M.C. (2020). Heat- and Ultrasound-Assisted Aqueous Extraction of Soluble Carbohydrates and Phenolics from Carob Kibbles of Variable Size and Source Material. Foods.

[26] Benzie I.F.F., Strain J.J. (1996). The Ferric Reducing Ability of Plasma (FRAP) as a Measure of “Antioxidant Power”: The FRAP Assay. Anal. Biochem..

[27] Rouphael Y., Colla G., Giordano M., El-Nakhel C., Kyriacou M.C., De Pascale S. (2017). Foliar applications of a legume-derived protein hydrolysate elicit dose-dependent increases of growth, leaf mineral composition, yield and fruit quality in two greenhouse tomato cultivars. Sci. Hortic..

[28] Bremner J.M. (1965). Total Nitrogen. Methods of Soil Analysis.

[29] Farag M.A., El-Kersh D.M. (2017). Volatiles profiling in *Ceratonia siliqua* (*Carob bean*) from Egypt and in response to roasting as analyzed via solid-phase microextraction coupled to chemometrics. J. Adv. Res..

[30] Krokou A., Stylianou M., Agapiou A. (2019). Assessing the volatile profile of carob tree (*Ceratonia siliqua* L.). Environ. Sci. Pollut. Res..

[31] Clewer A.G., Scarisbrick D.H. (2013). Practical Statistics and Experimental Design for Plant and Crop Science.

[32] Tetik N., Turhan I., Oziyci H.R., Gubbuk H., Karhan M., Ercisli S. (2011). Physical and chemical characterization of *Ceratonia siliqua* L. germplasm in Turkey. Sci. Hortic..

[33] El Batal H., Hasib A., Ouatmane A., Boulli A., Dehbi F., Jaouad A.J.J.o.M., Science E. (2013). Yield and composition of carob bean gum produced from different Moroccan populations of carob (*Ceratonia siliqua* L.). J. Mater. Environ. Sci..

[34] Farag M.A., El-Kersh D.M., Ehrlich A., Choucry M.A., El-Seedi H., Frolov A., Wessjohann L.A. (2019). Variation in *Ceratonia siliqua* pod metabolome in context of its different geographical origin, ripening stage and roasting process. Food Chem..

[35] Shakoor A., Zhang C., Xie J., Yang X. (2022). Maillard reaction chemistry in formation of critical intermediates and flavour compounds and their antioxidant properties. Food Chem..

[36] Rupasinghe H.V. (2008). The role of polyphenols in quality, postharvest handling, and processing of fruits. Postharvest Biology Technology of Fruits, Vegetables and Flowers.

[37] Krokou A., Kokkinofta R., Stylianou M., Agapiou A. (2020). Decoding carob flavor aroma using HS–SPME–GC–MS and chemometrics. Eur. Food Res. Technol..

[38] Racolta E., Tofana M., Muresan C., Socaci C., Florin G., Vlad M. (2014). Volatile compounds and sensory evaluation of spreadable creams based on roasted sunflower kernels and cocoa or carob powder. J Bull UASVM Food Sci Technol.

[39] El Hadi M.A.M., Zhang F.-J., Wu F.-F., Zhou C.-H., Tao J. (2013). Advances in Fruit Aroma Volatile Research. Molecules.

[40] McGorrin R.J. (2011). The Significance of Volatile Sulfur Compounds in Food Flavors. Volatile Sulfur Compounds in Food.

